# Right to Life or Right to Die in Advanced Dementia: Physician-Assisted Dying

**DOI:** 10.3389/fpsyt.2020.622446

**Published:** 2021-01-21

**Authors:** Jitender Jakhar, Saaniya Ambreen, Shiv Prasad

**Affiliations:** ^1^Department of Psychiatry, Govind Ballabh Pant Institute of Postgraduate Medical Education and Research, University of Delhi, New Delhi, India; ^2^Faculty of Medical Sciences, Lady Hardinge Medical College, University of Delhi, New Delhi, India

**Keywords:** psychiatry, ethical, burden, advanced dementia, physician assisted dying

“A mind that could be so alive one moment with thought and feeling building toward the next step and then someone erases the blackboard. It's all gone and I can't even reconstruct what the topic was. It's just gone. And I sit with the dark, the blank.” As the eminent American psychologist, Sandra Bem mused so eloquently in her writings, immediately after receiving a diagnosis of Alzheimer's (1). This anecdote very poignantly summarizes the harrowing effect dementia has on the individual person. There have been massive improvements in the health sector over the previous decades, and as a consequence people have been able to live longer and healthier lives. However, concurrently, there has also been an increase in the cases of non-communicable diseases, which includes dementia. In 2015 itself, dementia affected 47 million people worldwide; corresponding to roughly 5% of the world's elderly population (2, 3). Extensive research has been done for early and moderate stage dementia, but severe dementia is relatively neglected with very few evidence-based treatment options available. The later stages are very important because they harbor unique characteristics and events that affect not only the lives of the patients, but also their caregivers. In advanced stages, patients suffer from gradual deterioration in cognitive ability and functional impairments, which imposes a heavy burden on the public health system and families. Both direct and indirect costs of institutionalization are staggeringly large (4). These factors require a need for revision of existing health advisory and the below-mentioned concerns should be appropriately addressed before devising further health policy.

1. Economic burden: Dementia poses a high economic burden and the estimated annual cost to society for dementia is US$604 billion. This figure illustrates the massive impact of dementia and its caregiving worldwide. The per-capita costs of dementia are higher in higher-income countries compared to low or middle-income countries, where the majority of people with dementia are currently living. Approximately 90% of global societal costs of dementia are currently incurred in these higher-income regions (5). This implies that the infrastructure for the formal social care of dementia is very inadequate in lower-income countries, and the burden of care falls primarily on unpaid caregivers, where informal care costs predominate. This, along with the lower average wages in these areas imposes a further strain on the caregivers.
2. Caregiver burden: Advanced dementia results in multiple functional disabilities that include the patient being unable to take care of their hygiene, or being unable to feed themselves, and various other behavioral issues. This implies that their carers have to take over practical tasks such as bathing and toileting which give rise to a more physical burden of care (6). Caring for individuals with dementia at home is often described as “enduring stress and frustration” (7). Furthermore, behaviors such as aggression, agitation, and nighttime wandering are more strongly associated with cognitive and depressive symptoms in caregivers (8, 9). Studies performed in middle-income countries found that there is a mild to moderate range of burden present upon the carers, suggesting that caregiving for dementia entails great stress on the key caregiver. This stress increases linearly with an increase upon the inevitable progression of cognitive and behavioral symptoms, as well as the duration of caring for the loved one. All this takes an immense toll on the caregiver, which can lead to depression and other illnesses (10). Family conflicts may also arise because of the caregiving situation itself or when long-standing unresolved family issues continue to spill over into the caregiver's experience (11). The use of psychotropics was also found to be higher among caregivers than non-caregivers, which further adds to the economic cost (12).
3. Behavioral and psychological symptoms: As the illness advances, there is a myriad of changes in the personality of the person as well, which is especially disturbing for their families. Their prevalence can be upto 90% at a given point in the course of their illness in those diagnosed with an advanced stage of dementia (13). They may become introverted and unconcerned. They may become hostile and aggressive, which might put them in danger or hinder with caretaking. Lack of judgment and poor impulse control may appear which is further distressing.
4. End of life care crisis: When a person with dementia is nearing their end, they exhibit various signs, which might help in identifying the end time. These include limited speech, dependency for everyday activities, eating less and swallowing difficulties, plus bowel and bladder incontinence (14). At the end stage, the person might start losing consciousness, present with terminal restlessness, and changes in the breathing pattern. All of this can be very distressing for patients as well as for loved ones (15). There is immense suffering on the part of the caregiver, beyond the burden imposed by caregiving. Dementia ravages what was known and loved about the person. Caring for someone with dementia and seeing these harrowing changes intimately reflect upon dementia as a kind of “living death,” as if the person once known so well is no longer there, even though their body is still living and breathing (16). There is a unique form of grief before physical death owing to the anticipated and present losses of personhood. Caregivers often have “anticipatory grief,” where the grief work is accomplished during the illness phase and is largely resolved by the time physical death occurs. Anticipatory grief in dementia caregiving is “real” grief and is equivalent in intensity to normal death-related grief (17).
5. Early Mortality: In advanced dementia, apart from the slow and unavoidable aspect of losing competency, there is also a high risk for mortality as well. A study report that the median survival rate in the advanced stage is 1.3 years and approximately half of the subjects died over an 18-month duration follow-up period owing to complications of pneumonia, eating difficulties, and fever. These complications accounted for a high 6-month mortality rate in advanced dementia (18).

## Current Status of Physician-Assisted Dying Globally (PAD)

The term euthanasia is derived from the Greek word meaning pleasant death. The question of ending a life in terminally ill patients has always been one of exploring murky waters, owing to the multi-faceted and complex themes it raises. Physician-assisted suicide is a controversial topic with a much-heated debate around different corners of the world, and different perspectives are offered both historically and presently. The definition of PAD implies that the patient self-administers medications prescribed by the physician with the explicit intention of ending their life to get relief from intractable suffering (19). In June 1997, the US Supreme Court unanimously ruled that there is neither a constitutional prohibition nor a constitutional right to euthanasia or PAD. The Netherlands became the first country to legalize euthanasia, albeit under strict regulations (20). The law specifies that the person must be enduring “unbearable and endless suffering” before undergoing euthanasia. While euthanasia is the only legal form of PAD in Colombia and Belgium. Physician-assisted suicide is the legal form in Switzerland and five regions of the United States (Oregon, Vermont, Washington, California, and Montana). Both are legally permitted in Australia, Victoria, Canada, Luxemburg, and the Netherlands (21). However, among middle-income countries, there is still controversy regarding its implication. Regulations in other countries that allow for euthanasia are mentioned below (22):

1. The Netherlands: It become the first country in the world to formulate an extensive framework for the implementation of euthanasia. In 2016, the Dutch also proposed the guidelines to carry out euthanasia in advanced dementia patients under strict conditions with a written request.
2. United States: On October 27th, 1997, Oregon became the first legalized state in the United States to enact the “Death with Dignity act” for assisted dying (23). Oregon was then followed by Washington in 2008, Vermont in 2013, and subsequent legalization was done in the states of California, Colorado, Hawaii, Maine, and New Jersey. All these states passed the “Death with dignity act” except Montana which allowed PAD but didn't explicitly enact the act. Litigation is in the process in other states also. In 2009, passive euthanasia was also declared legal in New Mexico.
3. Belgium: The physician should convey the decision to perform euthanasia or PAS to a review committee, which assesses the report and may ask for verbal or written testimony. In 2014, Belgium also formulated a special law for euthanasia of terminally ill children. However, the child must ask for the procedure and verify that they understand what will happen.
4. Luxembourg: In 2009, Luxembourg legalized euthanasia as well. However, the law excludes minors and applies to anyone who is in a “hopeless medical condition.”
5. Switzerland: This country allows for assisted suicide by patients themselves. This has led to the curious phenomenon of “suicide tourism” wherein people come from other countries to carry out the act of suicide. This is also the only country where there is the formulation of a protocol for assisted suicide in individuals who suffer from some psychiatric illness.
6. Canada: In Canada, the bill was passed on 17th June 2016 and titled MAID (Medical Assistance in Dying). PAD was legalized throughout the country and in special cases; patients with advanced-stage dementia were also considered to be eligible.
7. Japan: Japan has no official definition and legal framework for euthanasia cases. The term “songenshi” is equivalent to death with dignity in Japan. The first case was noted in 1949, after that, there were six cases of mercy killing, with two prominent cases that received special attention: Yokohama court (1995) and the Kyoto court (1996) (24).
8. China: According to article 232 and 233 of Criminal Law of the People's Republic of China, PAD is illegal in china. Ethicists both oppose and defend it. Currently, the National People's Congress has not made any definitive decision regarding euthanasia (25).
9. Other countries: Over the years, there have been landmark cases concerning PAD in other countries where it is still illegal, but which nevertheless resulted in the heated debate by a variety of scholars, ethicists, lawyers, and doctors. These include the cases of Marie Fleming in Ireland and Lucio Magri in Italy.

### Indian Context

Only a few European countries have been successful in drafting legislature on euthanasia and assisted dying. India has also had a long history of grappling with the same. According to the Indian Penal code 1860, ending someone's life through any mode is a criminal offense altogether under Section 302 (punishment for murder) and Section 304 (punishment for culpable homicide not amounting to murder) (26). The first mention of any legislature on the same was proposed in the Euthanasia (Regulation) Bill, 2002 “to provide compassionate termination of the life of persons completely and permanently invalid or bed-ridden by the incurable disease” (27). However, the Parliament refused to legislate it. In 2005, the Guidelines by the Indian Society of Critical care Medicine (ISCCM) architected some key recommendations to address the issue. Upon request by the ISCCM, the 196th Report of the Law Commission of India drafted a bill titled, “Medical treatment to terminally ill patients” which focused specifically on the concept of passive euthanasia. However, this was rejected by the Government of India owing to its concerns regarding their doubts on it amounting to “intentional killing” and was a way of legalizing suicide (28) However, following the landmark case of Aruna Shanbaug, who was in a vegetative state and passed away from pneumonia in 2015 after being in a coma for 42 years, India legally allowed for passive euthanasia, which involves the withdrawal of treatment or food that would allow the patient to live. A cross-sectional study to assess the personal perception and attitude of Indian doctors about euthanasia found that the majority (69.3%) were found to be in support, with relief from unbearable pain and suffering being the most common reason for those who indicated their support (80.3%). Doubts regarding the misuse of euthanasia were the most common reason (66.2%) among those who advocated against euthanasia. It was also found that doctors from medical, surgical specialties, and pediatricians were more in support of euthanasia as compared to those practicing obstetrics/gynecology (29).

## Patient's Perspective

When patients are at their life's end and suffering from terminal illnesses like dementia, they frequently express the desire to not live any further. Life is often viewed as meaningless and they often express the opinion that they are held captive under their mental prisons. They often consider themselves to be a burden on their family members. As dementia progresses, all their activity of daily living and instrumental activities get impaired. This loss of personal autonomy results in guilt and shame among patients. This raises important ethical questions in mind: If people have the right to live, then why can they not also have the right to die, especially when they are suffering immensely under such unbearable and terminal conditions? Why can't we respect the right of the person to die at home with dignity, rather than prolong their suffering? (30). This becomes even more pertinent in advanced cases of dementia, where patients lost their capacity to make decisions. Patients diagnosed with dementia may carry out a written request detailing the circumstances of how they wish to die beforehand, rather than waiting for the advanced stages where they will no longer be able to express their will. Consistent with these changing views about the right to control the circumstances of one's life and death, Dutch laws allow for the formulation of an advance directive written by the patient in a cognitively lucid state, which is to be prepared before the patient may no longer be able to express themselves through direct communication. It is wise to consider that the patient's request to die may be an effort to assert their authority over life, even if this control is illusory and paradoxical. This might be preferred to forcing themselves upon society and their caregivers as a burden during advanced stages of dementia. Patients may prefer dying with dignity and in control of their end (31). A study to explore the personal experience of terminally ill patients found that the majority were in support of assisted suicide, and the most commonly cited reasons were anticipated pain, fear of indignity, and a loss of control (32). In another study among palliative care patients reported that roughly a quarter (29%) were in favor of euthanasia, one-fifth against (20%), while the majority were ambivalent (51%). Those in favor argued that suffering so intensely made life meaningless on a personal level, or had fears of frailty and loss of independence, and doubts regarding the provision of continuing help and communication (33).

## Psychiatrist's Perspective

For psychiatrists, this is a double-edged sword. Even though it is the moral and ethical obligation of psychiatrists to protect life, it is also equally imperative to maintain a state of psychological well-being, especially as the patient faces their end. The request for PAD in the presence of a psychiatric disorder or advanced dementia is an area of controversy. In the former case, the patient often reports the presence of depressive cognition, with or without severe morbidity; and in the latter; the patient loses their decision-making capacity as a part of the disease process itself. These issues make it more difficult for the team while considering the request of PAD in neuro-psychiatric patients (34). This difficulty was highlighted in a cross-sectional study of 2,269 Dutch physicians where the majority of physicians reported that they can conceive of granting a request for assisted dying in patients with cancer (85%), or another physical illness (82%), but only around one-third of physicians found it conceivable to provide PAD in patients with a psychiatric diagnosis (34%), early dementia (40%) or advanced dementia (~30%). This finding also implies that physicians are less likely to consider psychosocial suffering as intolerable as compared to physical suffering and which might in part be due to not having complete insight into their suffering (35). Patients with depressive disorder or any treatment-resistant psychiatric disorder are found to be more likely to put forward the request for physician-assisted dying, hence the early involvement of a psychiatrist in the team and adequate treatment of any Axis I diagnosis will aid the team in better decision-making while considering a request. Psychiatrists have a potential role in exploring and understanding patient modifiable or non-modifiable psychosocial factors. Besides assessing the mental capacity of patients for decision-making, they can also provide brief psychological support to the primary physician, who deals with the moral dilemma of the right to decide whether to take life, a province where God prevails.

## Community Perspective

A study in Finland to assess the attitude of the general public in Finland toward active voluntary euthanasia, passive euthanasia and physician-assisted suicide reported that approximately half of the general public supported euthanasia in a special situation, and around two-third population accepted passive euthanasia as a mode for the termination of life in dementia patients (36). Contrasted with their European counterparts, the African communities are more opposed to euthanasia. Surprisingly, a study to assess the cultural perspective on euthanasia through ethnocultural profile among 120 participants (African, Coloreds, and Europeans) found no statistically significant difference in attitudes between the various groups. However, older participants were found to favor euthanasia (37). Prolonged caregiving is usually associated with higher levels of perceived stress. However, appraisals and personal resources play a vital role and influence one's reactions to stressful events. An individual with higher personal resources will more likely appraise chronic stress as less taxing, thus decreasing the probability of negative physical and psychological consequences. Religion, which is a formal institution in the community, and spirituality, which is more elusive and personal in nature, help an individual cope with stressors. In Madrid and Spain, a survey of 128 dementia caregivers founded decreased anger in those who held stronger belief in religion and spirituality, possibly due to religion offering higher social support, and spirituality and religion both providing more individual meaning to these stressful situations, as a consequence of cognitive restructuring of these events. Hence, the caregiver feels less adrift (38).

## The Moral Dilemma of Professionals

Medical ethical guidelines commit each professional to four basic prima facie principles: beneficence, non-maleficence, autonomy, and justice when evaluating the merits and demerits of any medical procedure. Therefore, respecting the autonomy of patients for their decision of PAD should be equally balanced by the intent of doing good or atleast doing no harm to the patients. There will always be an inner conscientious objection to assisting those who choose to end their life, on account of the professional obligation of beneficence, but this has to be balanced equally by respecting patient autonomy. Significant impact on how the community perceives the physician's role can be achieved by engaging in ethical behavior and “doing the right thing” for patients (39). General physicians and other healthcare professionals working with patients of severe dementia face another conundrum with the prospect of permanent disability brought on by the disease toward the end of life. Choosing between seeing patients endure endless suffering or giving them a prescription of lethal medication to end it draws heavily on the mental well-being of the doctor. Life is considered to be sacred and each physician must protect human life, but concurrently, physicians should strive to preserve individual dignity and protect the patients from suffering the daily onslaughts brought on by the end of life. When physicians are no longer able to achieve the overall goals of care, it is more humane to adhere to the wishes of the patient or the proxy decision-maker rather than prolonging their misery further. Adherence to the dying's right to self-determination (autonomy) is central to ethical care at the end of life (40). Further contrasting views of the opponents and proponents are mentioned in Table 1.

**Table 1:** Contrasting Perspective on PAD.

**Patient Perspective**	**Doctor Perspective**
**Proponents** • Patient autonomy and right to end life in a dignified way • In the absence of adequate treatment or cure, the latter half of life shouldn't be colored by disability and dependency • Wish to assuage their own enduring as well as the enduring of their loved ones • Lack of purpose to survive further with a poor cognitive reserve and no clear scope of improvement • Loss of social relationship and respect of loved one's overdue course of dementia can be very horrifying • Having control over life during the crisis phase of life can install positive emotion and satisfaction, hence better quality of life in leftover time. • The majority of public opinion favors PAD and should respect democracy and social justice.	**Proponents** • Ethically, it's unjust to delay someone's end by the range of intervention without improving their quality of life. • Existing law needs more sympathy and benevolence toward patients, denying the doctors the choice of finishing the patient's desolation and agony. • A few patients resort to suicide or more demeaning method e.g., shots or hanging • Can lead to better manpower utilization and have a better investment of time on the care of other patients • With restricted medical care assets and health care resources, assisted dying can be a less-exorbitant option in contrast to palliative care • Caregiver burden and stress further impairs the family functioning • High 6-month mortality rate and complication in advanced dementia
**Opponents** • Fear of abuse as few vulnerable advanced dementia patients may still be competent, but judged by caregivers as “not worth living” e.g., Physical disability • Only God has the right to control death and life and to end someone life, even at their solicitation, would disregard divine law • Choice of PAD may not be a free decision by the patient, yet might be constrained by socially restricting choices • Restricted understanding about dementia course and perceived disability and burden on loved ones in future years, might trigger an early request • The longing to bite the dust to dodge continued dementia might stem from prejudice, ignorance, inability to acknowledge adaptation, and dread of helpless nursing home consideration • There is no inherent good distinction between assisting death or killing someone, especially when the result is the equivalent	**Opponents** • PAD is fundamentally conflicting with the doctor role of saving lives and will breach the doctor-patient relationship and public faith in medicine • Professionally, PAD ought not to be an alternative until all patients have equal access to palliative care. • If both patients and family members get adequate psychosocial support and high-quality palliative care, they might not have any further desire to end life. • Pressure and untimely request from the caregiver in the condition of burden of care • Psychologically “Phenomena of adaptation”-Grief about dementia diagnosis and ignorance about perspective adaptation in due course of time might trigger an early request • Difficulty in patient-doctor communication in later stages will put the question on the validity of the patient request • Euthanasia request can be professionally taxing and install negative emotion of disappointment, moral distress, and helpless

## Proposed Guidelines and Evidence

Before any serious discussion on PAD requests, appropriate documentation and evaluation should be done. The Dutch Act states that euthanasia and physician-assisted suicide are not punishable if the attending physician acts in accordance with the law. Acting with strict adherence to the laws (41), we propose the following steps to be followed while considering any request for PAD: (1) Voluntary request from the patient (2) Advanced directive with a nominated representative request, especially in the advanced stage of dementia (3) Referral of the case to an established committee (4) Assessment of decision-making capacity (5) Screening for psychiatric disorders (6) Treatment of comorbid- Axis 1 psychiatric diagnosis, if any (7) Algorithm-based decision making - about the current situation and alternative options (8) Documentation of unbearable suffering and lack of reasonable options (9) Family involvement in the process (10) Submission of the report to the committee and applied method of ending life, and (11) Termination of life with due care. To check for adherence to strict criteria, the Netherlands reviewed 158 cases of PAD and euthanasia wherein they found good adherence to the established rules, and the patient request was well considered. The majority of the cases requested PAD due to incapacitating physical symptoms (62%), while others requested due to loss of functional capacity (33%), dependency on others for basic needs (28%), and gradual deterioration of health (15%) (42). Also, the perception of doctors and the general public toward the legalization of PAD is not well studied. An online survey was conducted on a sample of 3,773 UK medical practitioners from various specialties -physicians, palliative medicine specialists, neurologists, specialists in geriatric medicine, and others (except those engaged in public health) – to assess their attitudes toward assisted dying. There was found to be lesser levels of support by doctors of all categories for assisted dying when compared with the general public. The opposition was found to be stronger among palliative medicine doctors and elderly care specialists (43). Religion was also found to be associated independently with opposition to assisted dying. However, those doctors who were in favor of assisted dying stressed the importance of the need for adequate end-of-life care provisions and expressed concerns about the risk of potential abuse and the need for safeguards against the same. Interestingly, another review of the attitude of the American public from 1970 onwards found a more favorable response to PAD as compared to physicians, and approximately two-thirds of the public supported its legalization. Around one-third supported voluntary active PAD giving due consideration to the patient finding their lives meaningless and considering themselves a burden on their family, even in the absence of physical suffering (44).

## Precautionary Approach in Advanced Dementia

While the Netherlands has been notable for being one of the first countries to have legalized euthanasia, they have also faced ethical and moral dilemmas when it comes to its implementation in patients with advanced dementia. Apart from the community expressing worries about the potential abuse of this provision, the technical aspects of the laws can also be challenging. This was reflected in a landmark case (45) in the early 2000s, where a Dutch lady in her 70s wrote an advanced euthanasia directive early in the course of her Alzheimer's dementia, fearing her prospects of losing her sense of “self” throughout the illness and the inevitable deterioration that it would entail. She wrote an Advanced Euthanasia Directive which detailed how she wished to die: “whenever I think the time is right,” and “when the quality of my life has become so poor, I would like my request for euthanasia to be honored.” Upon her admission into a nursing facility, her husband requested the physician to perform euthanasia and the same was done. The Committee found an issue with the deception that the physician performed by adding a sedative to the patient's morning breakfast and the implementation of the procedure despite the patient physically resisting it. On both these counts, the Committee felt that the physician had not honored the dignity of the patient and had used surreptitious means to perform the procedure. In this case, the Dutch Review Committee found that the physician performing euthanasia failed to follow the specific criteria that were laid down, and this resulted in a criminal investigation being initiated against the physician. This case highlighted the difficulty of applying PAD on persons suffering from dementia, and also raised various philosophical and ethical dilemmas regarding the nature of the illness itself: at what point is it ethical for the “previous” self to make decisions for the “present” (demented) self? Moreover, the “previous” self might have underestimated the “present” self's ability to cope with the challenges—both physical and psychosocial—thrown by dementia, and so is it correct for the “previous” self to be making these decisions preemptively? Also, evidence states that decision-making might be hampered even early in the course of the illness, so is an advanced euthanasia directive truly a document that was formulated in the cognitively lucid state?

## Decision-Making and Advanced Directive

Decision-making capacity is a statutory requirement to give an informed decision about medical care. In practically all countries where PAD is legitimate, there is a prerequisite to assess the mental competency of the patient at the time of initiating their request for euthanasia. This is done to ensure the right applicability of law for those who are in need and also to make sure that they have the cognitive ability to decide the time to die when they reach the phase of unbearable suffering. This implies that patients with advanced dementia will be ruled out automatically as a candidate, even if they are terminally ill and incapacitated. Clinical assessment of competency in dementia patients should be a thorough comprehensive evaluation by neuropsychological testing, clinical and diagnostic interview, functional ability assessment, and a review of the legal standards in that existing country (46). One way to address this necessity of contemporary competence is by carefully writing an “advanced euthanasia directive” beforehand when decision-making capacity is still intact and satisfies the criteria of voluntariness. In June 2011, the Royal Dutch Medical Association (KNMG) proposed the expansion of Euthanasia Act 2002 to a special class of dementia, which requires extreme care and sensitivity, while implementing criteria of due care. The law stipulates that physicians should act following advanced directives, in cases where individuals are no more competent and may replace the existing contemporary concurrent personal request for euthanasia (47). To address this issue, Megan Wright proposed the concept of the Supported decision-making model (SDM) where a patient's autonomy can be promoted by relying on others while making an important decision and suitable legal documentation about these pre-existing decision-making relationships (48). Another alternative for this is the enactment of the “Adult Guardianship and Co-decision-making act” (49). This act intends to secure the interests of patients by legalizing the role of guardianship and co-decision-makers role if dementia patients have difficulty in decision making.

## Numbers of Cases

In patients with dementia, special caution needs to be exercised while applying the criteria of due care for PAD requests. The number of requests made for euthanasia significantly outweigh the number granted, as not every request satisfies the criteria of due care. Dutch law makes PAD accessible to two categories in patients with dementia. One category covers early-stage dementia patients, who have preserved decision-making capacity and can put forward their concurrent request for euthanasia. The other category includes those patients with advanced dementia who have previously submitted an advanced request through a written directive. Despite the fact, that dementia requests constitute only a small percentage of overall Dutch PAD requests, the number has significantly increased since 2011 (50). Most concurrent requests were made in the early phase of dementia when the individuals are competent and but from 2011 onwards, a small number of requests were granted PAD based on the advanced written directive. Euthanasia requests based on advanced directives are very rare (A total of seven in the last 3 years). Between 2016 and 2011, they were constituted of small numbers with no clear cut demarcation, and before 2011, none of the reported cases mentioned advanced directives. In 2019, The Dutch Regional Euthanasia Review Committees received a total of 6,361 notifications out of which 160 were early staged dementia and two were advanced staged dementia. There is a 3.8% increment in the euthanasia notification compared to 6,126 notifications in 2018 and a decrement by 4.4% compared to 6,585 notifications in the year 2017. Between the years 2002–2008, dementia requests were clubbed under the neurological disorder category (51). The number of reported euthanasia cases as per Dutch Annual report notification/year is shown in Table 2.

**Table 2:** Dutch Annual Euthanasia notifications/year.

**Year**		**2019**	**2018**	**2017**	**2016**	**2015**	**2014**	**2013**	**2012**	**2011**	**2010**	**2009**
Total Euthanasia Notification		6,361	6,126	6,585	6,091	5,516	5,306	4,829	4,188	3,695	3,136	2,636
Total number of dementia cases	Early staged dementia	160	144	166	141	109	81	97	42	49	25	12
	Advanced staged dementia	2	2	3	*	*	*	*	*	*	*	*

* *Before 2016, advanced dementia notification are not mentioned separately*.

A review of 75 published case reports of the Dutch regional euthanasia review board between 2011 and 2018 (total of 834 dementia notification), found that 59 requests were concurrent and 16 requests were advance, through a written directive. The majorities of them were women (53%) and had Alzheimer's diagnosis (48%). An advance request had a longer duration of 5.3 years diagnosed as dementia, before receiving euthanasia than 2.3 years duration in the concurrent request (51). In other countries also, PAD is still not so common in actual practice for the dementia population and a review of 179 reported cases in Belgium found that the proportion of dementia/psychiatric diagnosis accounts for 0.5% of all euthanasia cases in the period (2002–2007) and increasing up to 3% of all cases in 2013 (52). Moreover, PAD is further very rare for advanced dementia in these countries.

## Conclusion

There consistently have been and will continue to be contentions among professionals regarding the topic of PAD. This dilemma becomes more complex when applied to patients who suffer from devastating and advanced stages of illness. With changing laws and wider access to information, there is a need to gather more research data for the arrival of an evidence-based approach. When such enactments are made, frameworks must be set up to appropriately watch that the law is not abused.

## Author Contributions

All the authors were involved in conceptualization and drafting of the manuscript and contributed to the article and approved the submitted version.

## Conflict of Interest

The authors declare that the research was conducted in the absence of any commercial or financial relationships that could be construed as a potential conflict of interest.
